# Perceived Barriers for Physical Activity in Overweight and Obese Adolescents and Their Association with Health Motivation

**DOI:** 10.34763/devperiodmed.20172103.248258

**Published:** 2017-10-28

**Authors:** Maria Jodkowska, Anna Oblacińska, Hanna Nałęcz, Joanna Mazur

**Affiliations:** 1 Department of Child and Adolescent Health, Institute of Mother and Child, Warsaw, Poland

**Keywords:** body mass index, BMI, physical activity, adolescents, body weight control, barriers, motivation, body mass index, BMI, aktywność fizyczna, nastolatki, kontrola masy ciała, bariery, motywacja

## Abstract

**The aim:**

to identify the barriers to engaging in physical activity that are perceived by adolescents with overweight and obesity, and to establish whether a correlation exists among the barriers they perceive and the health-oriented motivation for undertaking physical activity.

**Material and methods:**

The study was conducted on a representative sample of 2,300 students aged 13-16 years. The data was collected through an anonymous survey. Young people were asked about their body weight and height, the barriers to physical activity and the health-related motives to engaging in it. The IOTF (International Obesity Task Force) standard by T. J. Cole was used to categorise overweight and obesity, while the PCA − Principal Component Analysis − to assess the motivation for physical activity. Logistic regression was used in the analyses of the correlations among the body weight, the level of health-oriented motivation and the occurrence of accumulated barriers to physical activity.

**Results:**

Overweight and obesity was found in 12.4% of the respondents; more often in boys (17.8%) than in girls (7.8%). The most frequently perceived barriers to undertaking physical activity among overweight adolescents include deficiencies in energy, time and support. Three barriers (lack of energy, skills and willpower), as well as the perception of several barriers occurring simultaneously, were reported more frequently by overweight students in comparison with their peers with a normal weight. Among the health-oriented motivation for physical activity in the group of adolescents with overweight and obesity, the most important one was the need to improve health, while the least important the need to look good. The excess of body weight turn out to be an important predictor of the perception of cumulative barriers to physical activity only among adolescents with a weak motivation to undertake physical activity [OR 2.51; CI (1.43-4.42), p<0.001].

**Conclusions:**

Young people with overweight and obesity, but with a strong health-oriented motivation, perceive barriers to physical activity similarly to their peers with a normal weight. Thus, motivation is a key element shaping the physical activity of overweight and obese young people by changing their perception of the barriers. In overcoming the barriers to physical activity in obese adolescents, one should aim to comprehensively reduce body weight and to support health-oriented motivation.

## Streszczenie

**Celem pracy:**

była identyfikacja barier w podejmowaniu aktywności fizycznej postrzeganych przez młodzież z nadwagą i otyłością oraz zbadanie czy istnieje związek pomiędzy postrzeganymi przez tę młodzież barierami, a ukierunkowaną na zdrowie motywacją do podejmowania aktywności fizycznej.

**Materiał i metody:**

Badanie przeprowadzono w reprezentatywnej grupie 2300 uczniów w wieku 13-16 lat. Dane zebrano metodą sondażu za pomocą anonimowej ankiety audytoryjnej. Młodzież pytano o ich masę i wysokość ciała, bariery w aktywności fizycznej oraz motywy zdrowotne jej podejmowania. Do klasyfikacji nadwagi i otyłości posłużono się Międzynarodowym Standardem IOTF (International Obesity Task Force). W ocenie motywów podejmowania aktywności fizycznej wykorzystano analizę czynnikową metodą głównych składowych (PCA – Principal Component Analysis). W analizach zależności między masą ciała oraz poziomem motywacji ukierunkowanej na zdrowie a występowaniem skumulowanych barier w podejmowaniu aktywności fizycznej, zastosowano metodę regresji logistycznej.

**Wyniki:**

Nadwaga i otyłość występowała u 12,4% badanych, częściej u chłopców (17,8%) niż u dziewcząt (7,8%). Do najczęściej postrzeganych barier w aktywności fizycznej u młodzieży z nadwagą należy brak energii, czasu oraz wsparcia. Trzy bariery (brak energii, umiejętności, silnej woli) oraz postrzeganie kilku barier jednocześnie istotnie częściej zgłaszała młodzież z nadwagą w porównaniu z rówieśnikami o prawidłowej masie ciała. Wśród zorientowanych na zdrowie motywów podejmowania aktywności fizycznej, w grupie młodzieży z nadwagą i otyłością najważniejsza była potrzeba poprawy zdrowia, natomiast najmniej ważna, potrzeba dobrego wyglądu. Nadmiar masy ciała okazał się istotnym predyktorem postrzegania kilku barier w aktywności fizycznej tylko u młodzieży o małym poziomie motywacji [OR 2,51; CI (1,43-4,42)].

**Wnioski:**

Młodzież z nadwagą i otyłością, ale o dużej motywacji ukierunkowanej na zdrowie postrzega bariery w aktywności fizycznej podobnie jak rówieśnicy z normalną masą ciała. Motywacja jest więc kluczowym elementem, który poprzez zmianę postrzegania barier, modeluje aktywność fizyczną młodzieży z nadwagą i otyłością. W pokonywaniu barier w aktywności fizycznej u młodzieży otyłej należy kompleksowo dążyć do redukcji masy ciała oraz wspierać ukierunkowaną na zdrowie motywację.

## Introduction

Both epidemiological studies and everyday observations of adolescent behaviours confirm the finding that adolescents with overweight and obesity are characterised by lower physical activity than their peers with normal weight. Moreover, overweight adolescents take part in PE classes and extracurricular sports less often [1, 2]. Unfortunately, despite evidence proving the benefits of physical activity for health and development (including obesity and atherosclerosis prevention), most of the children and adolescents are not physically active on a recommended level or they prefer a sedentary lifestyle [3, 4]; alternatively, both reasons occur simultaneously resulting in an accumulation of factors which have a negative impact on health and development [5].

It is, therefore, important to investigate the reasons why overweight adolescents are less active than their peers with normal weight.

The barriers perceived by young people probably have a potential influence on physical activity. Some barriers may have a stronger negative impact on engaging in physical activity than others; they may also be more specific for adolescents with excess body mass. These are primarily the so-called internal barriers resulting from low self-esteem and self-limitations, such as lack of skills and lack of energy. Overweight might have a negative impact on achieving the milestones in child development and shaping the basic motor skills in young people. It was demonstrated that obesity significantly hinders some motor activities, such as jumping, climbing or squatting. Research has also shown an inverse relationship between cardiorespiratory fitness and obesity level [6]. In some types of aerobic activities, such as running, overweight children and adolescents are unable to cover the same distance as their peers with a normal weight, as they become tired more quickly. Because of the functional and structural limitations related to obesity among children and adolescents, it is also obvious that the health-related quality of life in this group is lower in comparison with their slim peers [7].

Motivation plays a significant role in maintaining physical activity. Understanding its role in pursuing an active lifestyle is the key to tackling the problem of an insufficient amount of physical activity. This problem largely affects overweight and obese children and adolescents [8]. Most of the studies which evaluate the physical activity of obese people fail to consider the psychological factors related to obesity. However, the self-determination theory (STD) by E. Deci and R. Ryan is increasingly often quoted as the theoretical basis for the studies conducted. It is used to identify the relation among undertaking and maintaining physical activity and psychological variables in obese people [9, 10]. The SDT explains the mutual influence of internal and external factors, as well as the natural human needs which have impact on human behaviour [11, 12].

According to Edmunds et al., more is needed to maintain an active lifestyle than just internal motivation; one also needs external factors, such as peer support or trends [13]. External motivation seems, however, to be key in undertaking physical activity [14, 15].

**Table I:** Barriers to physical activity − items and the type of barrier. Tabela I Stwierdzenia dotyczące barier w podejmowaniu aktywności fizycznej oraz rodzaj bariery.

	Item Stwierdzenie	Barrier type Rodzaj bariery
1	I’m just too tired after school to get any exercise *Jestem zbyt zmęczony/a po lekcjach, aby zajmować się aktywnością fizyczną*	Lack of energy *Brak energii*
2	It’s easier for me to find an excuse not to exercise than to go out and do something *Łatwiej znaleźć mi wytłumaczenie, dlaczego nie ćwiczę, niż wyjść i poruszać się*	Lack of willpower *Brak silnej woli*
3	My free time during the day is too short to include exercise *Mam zbyt mało wolnego czasu, aby znalazły się w nim zajęcia związane z aktywnością fizyczną*	Lack of time *Brak czasu*
4	My usual social activities with family or friends do not include physical activity *Moje zwykłe zajęcia w rodzinie i z przyjaciółmi nie obejmują aktywności fizycznej*	Lack of support *Brak wsparcia*
5	I’m not good enough at any physical activity to make it fun *Nie jestem dostatecznie dobry/a w zajęciach sportowych i ćwiczeniach fizycznych, aby sprawiały mi one radość*	Lack of skills *Brak umiejętności*

The most important factors motivating overweight and obese adolescents to physical activity include mixed and external motives occurring under the influence of the social environment, such as the need to lose weight or to improve physical fitness. At the same time, for adolescents with normal weight, internal motives, such as pleasure and fun, are essential [16, 17, 18].

**The aim of the study** was to analyse the barriers to engaging in physical activity that are reported by adolescents with excessive body mass, and determine whether they perceive those barriers similarly to their peers with normal weight? Moreover, the aim was to establish whether relationships exist among the perceived barriers and the health-oriented motivation to physical activity.

## Material and methods

The study was conducted in 2013 as part of the project funded by the Ministry of Sports and Tourism in Poland in 2013, on a representative sample of 3,346 students aged 10-16 (including 1,587 boys) 1Analysis were conducted within the statutory activity of the Institute of Mother and Child, project No 510-20-65. The data was collected through an anonymous survey among students from 163 classes from 68 randomly selected schools. Questions about the barriers to physical activity and the motivation to undertake it were answered by adolescents aged 13-16 (median age: 14.9 years; SD=1.2), N= 2,300 persons (1,259 girls and 1,041 boys). The study was conducted in accordance with the Declaration of Helsinki, and were approved by the Bioethical Committee of the Institute of Mother and Child, Warsaw, Poland (No. 20/2013).

### The investigated variables and their indicators Body mass index

Participants answered the questions about their weight and height. The data was used to calculate the BMI index. In the classification of overweight and obesity, the IOTF (International Obesity Task Force) standards, developed by T. J. Cole were used [19].

### The barriers to undertaking physical activity

The test Barriers to being active. What keeps you from being more active? was used to examine the barriers to undertaking physical activity. This tool is devised and distributed by the Centers for Disease Control and Prevention (CDC) [20]. It is composed of 21 statements, which comprise seven barriers: lack of energy, time, social support, willpower, skills, lack of sports facilities and fear of injury. The test was adapted for the Polish language and culture. After piloting, five statements on the scale were selected for further study; they were characterised by psychometrical cohesion. They comprised five out of seven of the areas of the original tool: lack of energy, time, social support, willpower, and skills.

Table I shows the barriers and their corresponding statements of the scale. Participants indicated how likely they were to say each of these statements using the 4-level Likert scale: very likely, somewhat likely, somewhat unlikely, very unlikely. In our sample the positive psychometric properties of this 5-item short scale were confirmed, and its internal consistency was evaluated using Cronbach alpha, and equaled 0.82. Principal component analysis revealed the presence of one component with eigenvalue exceeding 1, explaining 58% of the variance.

**Table II:** Characteristics of particitants (N=2300) by gender, grade and body mass (%). Tabela II Charakterystyka badanej młodzieży (N=2300) według płci, klasy oraz nadmiaru masy ciała (%).

Gender/Grade *Płeć/Klasa*	%
Boys *Chłopcy*	45.3
Junior high school grade I and II *Klasa I i II gimnazjum*	
Boys Chłopcy	49.5
Junior high school grade III and senior high grade III *Klasa III gimnazjum i I kl. ponadgimnazjalna*	
Boys *Chłopcy*	41.0
**Overweight and obesity *Nadmiar masy ciała***	%
Total *Ogółem*	12.4
Boys *Chłopcy*	17.8
Girls *Dziewczęta*	7.8
p<0,001	
**Junior high school grade I and II *Klasa I i II gimnazjum***	%
Total *Ogółem*	13.8
Boys *Chłopcy*	18.1
Girls *Dziewczęta*	9.7
p<0,001	
**Junior high school grade III and senior high grade III *Klasa III gimnazjum i I kl. ponadgimnazjalna***	%
Total *Ogółem*	10.9
Boys *Chłopcy*	17.4
Girls *Dziewczęta*	6.2
p < 0,001	

The evaluation of barriers to physical activity was done by summarizing the responses of participants who answered very likely and somewhat likely to each statement as presence of barrier, and the remaining two answers (somewhat unlikely, very unlikely) as lack of barrier. The accumulated occurrence of the barriers to undertaking physical activity was also estimated. It was assumed that the indicator represents young people’s perception of two or more out of the five possible barriers.

**Table III:** Perceived barriers in physical activity across BMI categories (%). Tabela III Postrzeganie aktywności fizycznej u młodzieży według kategorii masy ciała (%).

Barrier type *Rodzaj bariery*	Total *Ogółem*		P	Boys *Chłopcy*		P	Girls *Dziewczęta*		P
	Overweight and obesity *Nadwaga i otyłość*	Normal body mass *Prawidłowa masa ciała*		Overweight and obesity *Nadwaga i otyłość*	Normal body mass *Prawidłowa masa ciała*		Overweight and obesity *Nadwaga i otyłość*	Normal body mass *Prawidłowa masa ciała*	
Lack ** of ** energy Brak energii	50.8	44.7	0.039	45.8	32.8	0.001	60.2	53.6	0.137
Lack ** of ** time Brak czasu	43.1	40.9	0.273	39.3	29.9	0.012	50.6	49.2	0.445
Lack ** of ** support Brak wsparcia	40.0	40.7	0.438	38.9	35.1	0.197	42.0	45.0	0.340
Lack ** of ** willpower Brak silnej woli **	38.7	32.2	0.024	32.7	25.1	0.027	50.0	37.5	0.015
** Lack ** of skills Brak umiejętności	36.8	23.2	0.000	30.1	19.7	0.003	49.4	25.7	0.000
Accumulated barriers (2-5 barriers) Kumulowanie barier *(2-5 barier)*	58.2	50.2	0.010	51.8	38.7	0.001	70.1	58.6	0.022

### The motives for engaging in physical activity

A full, three-dimensional scale of the motives for undertaking physical activity was adapted for the Polish version in the 2005/2006 round of the HBSC survey; a detailed description can be found in a separate publication [21]. This paper includes one factor of the full scale health-oriented motivation, which comprised the following statements: to improve my health; to get in good shape; to look good; to control my weight; to be good at sport. The 5-item scale proved to have good psychometric properties: one factor explained 53.5% of the variability of the scale; its reliability measured with Cronbach’s alpha was 0.77. Adolescents assessed the importance of the motives listed on a 3-point scale, from 0 (not important) to 2 (very important). The higher the score on a scale, the stronger the motivation to undertake physical activity.

**Table IV:** Health motivation for adolescents PA across BMI categories - mean and standard deviation. Tabela IV. Motywy podejmowania aktywności fizycznej zorientowane na zdrowie u młodzieży według kategorii masy ciała - średnia i odchylenie standardowe.

Motives *Motywy*	Total *Ogółem*				P	Boys Chłopcy				P	Girls *Dziewczęta*				P
	Overweight and obesity *Nadwaga i otyłość*		Normal body mass *Prawidłowa masa ciała*			Overweight and obesity *Nadwaga i otyłość*		Normal body mass *Prawidłowa masa ciała*			Overweight and obesity *Nadwaga i otyłość*		Normal body mass *Prawidłowa masa ciała*		
	M	SD	M	SD		M	SD	M	SD		M	SD	M	SD	
to improve my health *dla poprawy zdrowia*	1.69	0.56	1.71	0.52	0.630	1.68	0.58	1.70	0.53	0.604	1.70	0.50	1.72	0.52	0.746
to get in good shape *żeby być w dobrej formie*	1.61	0.62	1.73	0.50	0.003	1.64	0.63	1.75	0.49	0.082	1.55	0.59	1.72	0.50	0.002
to be good at sport *żeby być sprawnym fizycznie*	1.59	0.61	1.69	0.53	0.009	1.62	0.62	1.73	0.51	0.038	1.52	0.61	1.66	0.54	0.019
to control my weight *żeby kontrolować masę ciała*	1.58	0.66	1.48	0.71	0.028	1.54	0.68	1.36	0.75	0.003	1.66	0.60	1.57	0.66	0.229
to look good *żeby dobrze wyglądać*	1.58	0.64	1.66	0.58	0.094	1.50	0.69	1.56	0.62	0.417	1.74	0.51	1.73	0.53	0.937

### Statistical analysis

The statistical analysis entailed descriptive statistics including: percentages, mean values and standard deviations of the variables analysed. Differences between groups were calculated using the chi^2^ test for frequency, nonparametric tests for means Mann-Whitney (M-W)

In the analysis of the motives for undertaking physical activity, the PCA (Principal Component Analysis) was used. The new variable − “health-oriented motivation” was subsequently divided on the basis of tertiles, as follows: weak, average, strong motivation to physical activity.

In the analyses of the relationships between body weight, the level of health-oriented motivation and the occurrence of accumulated (2 or more) barriers to physical activity, adjusted for gender and age, logistic regression was used; for all of the analyses p<0.05 was adopted as the sufficient significance level. The software used was SPSS v. 19.0.

## Results

The characteristics of participants are shown in table II. Overweight and obesity occurred in one of every eight people; twice as often in boys as in girls (p<0.001). In the younger age group, adolescents with excessive body mass comprised 18.1% and 9.7% boys and girls, respectively (p<0.001). In the older age group, there were twice as many boys with excessive body mass as their female peers (p<0.001).

### Perceived barriers to physical activity in adolescents with excessive body mass

The factors most frequently perceived as barriers to undertaking physical activity by adolescents with excessive body mass were: lack of energy, time and social support (reported by 40-51% of the respondents) (tab. III).

Three out of five of the analysed barriers were, indeed, more often reported by adolescents with excessive body mass in comparison to their normal weight peers. The greatest differences were noted in the barrier “lack of skills” (36.8% vs. 23.2%, p=0.000). No statistically important differences were found in the occurrence of the barrier “lack of social support” (in general and in both genders) and “lack of time” (in general and in girls). Greater differences were noted in the perception of the barriers in overweight and obese boys in comparison with their normal weight peers (4 out of 5 barriers) than in girls (significant differences for 2 out of 5 barriers). The analysis conducted showed that young people with excessive body weight did indeed perceive several barriers to physical activity simultaneously (accumulated barriers). It was found that over half of the boys and 70% of the girls in that group perceived at least two out of five of the investigated barriers.

### The motives for engaging in health-oriented physical activity

In the general sample of adolescents with overweight and obesity, the most important reason for engaging in health-oriented physical activity, was the need to improve one’s health, while the least important one was the need to look good (tab. IV).

In gender analysis, it was noted that in girls with excessive body mass the need to look good was the strongest motive to physical activity. The most important motive among adolescents with normal weight was to be good at sport, while to control my weight proved to be the least important one. In comparison with peers with normal weight, adolescents with overweight and obesity were characterised by lower motivation to get in good shape (p=0.003) and to be fit (p=0.009). However, they had stronger motivation to control my weight, though a statistically important correlation was found only in boys (p=0.003).

**Fig. 1:**
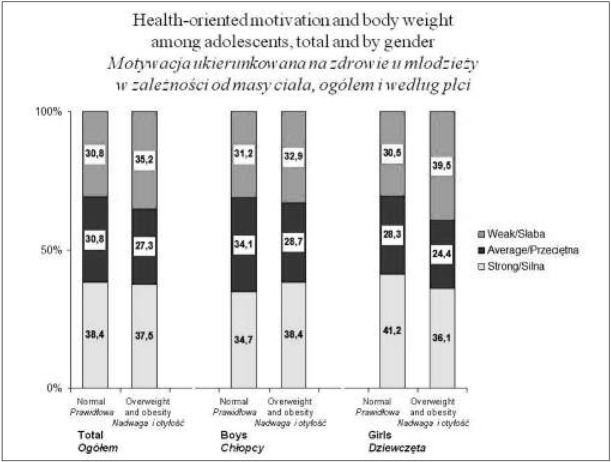
Health motivation for adolescents PA across BMI categories and gender (%). Ryc. 1. Motywacja do aktywności fizycznej ukierunkowana na zdrowie u młodzieży z różną masą ciała, ogółem i według płci (%).

No statistically significant differences were found regarding health-oriented motivation among young people with normal weight and their peers with overweight and obesity. Attention should be drawn to the tendency occurring in girls with excessive body weight: there was a greater percentage of persons with weak motivation for health in comparison with their peers with normal weight (39.5% vs. 30.5%) and analogically a lower percentage of those with strong motivation (36.1% vs. 41.2%) (fig. 1). Such a correlation was not found in boys.

**Table V:** Multidimensional logistic regression modeling results with estimated risk (OR) of adolescents’ cumulated PA barriers perception. Tabela V Wyniki wielowymiarowego modelu regresji logistycznej z oszacowanym ryzykiem (na podstawie ilorazu szans) postrzegania przez młodzież skumulowanych barier w aktywności fizycznej.

Variables *Zmienne*	B	p	OR	95% (OR)	
				Coefficient interval *Przedział ufności*	
Gender *Płeć* Boys (ref) *Chłopcy (ref)*				Upper limit *Dolna granica*	Lower limit *Górna granica*
Girls *Dziewczęta*	0.845	0.000	2.33	1.92	2.82
Age *Wiek* 13,8 lat (ref)					
15,8 lat	0.516	0.000	1.68	1.39	2.02
BMI categories *Kategorie BMI* Normal body mass (ref) *Prawidłowa masa ciała (ref)*					
Overweight *Nadwaga i otyłość* and obesity	0.542	0.000	1.72	1.29	2.30
Health-oriented motivation *Motywacja dla zdrowia* Strong (ref) *Silna (ref)*					
Average *Przeciętna*	0.192	0.090	1.21	0.97	1.51
Weak *Słaba*	1.085	0.000	2.96	2.36	3.72
Constant (β) *Stała (*β*)*	1.139	0.000	0.32		

**Table VI:** Multidimensional logistic regression modeling results with estimated risk (OR) of adolescents' cumulative PA barriers perception within the health motivation groups. Tabela VI. Wyniki wielowymiarowego modelu regresji logistycznej z oszacowanym ryzykiem (na podstawie ilorazu szans OR) postrzegania przez młodzież skumulowanych barier w aktywności fizycznej w różnym zależności od poziomu motywacji ukierunkowanej na zdrowie.

Health-oriented motivation *Motywacja dla zdrowia*	Weak motivation *Motywacja słaba*			Average motivation *Motywacja przeciętna*			Strong motivation *Motywacja silna*		
	B	P	OR (95% Cl)	B	P	OR (95% Cl)	B	P	OR (95% Cl)
Gender *Płeć* Boys (ref) *Chłopcy (ref)*									
Girls *Dziewczeta*	0.915	0.000	2.50 (1.76-3.55)	0.976	0.000	2.65 (1.89-3.73)	0.678	0.000	1.97 (1.45-2.68)
Age *Wiek* 13,8 lat (ref)									
15,8 lat	0.275	0.121	1.32 (0.93-1.86)	0.752	0.000	2.12 (1.52-2.97)	0.521	0.001	1.68 (1.25-2.26)
BMI categories Kategorie BMI Normal body mass *Prawidłowa masa (ref)*									
Overweight *and obesity Nadwaga i otyłość*	0.920	0.001	2.51 (1.43-4.42)	0.397	0.143	1.49 (0.87-2.53)	0.370	0.116	1.45 (0.91-2.30)
Constant (β) *Stała (β)*	-.005	0.976	0.995	-1.116	0.000	0.327	-1.018	0.000	0.361

### Perceived barriers and their relationships with body weight and the level of motivation for health-oriented physical activity

The assessed model of logistic regression showed that a greater risk of perceiving accumulated barriers to physical activity occurred in girls, in older adolescents and in people with excessive body weight (tab. V). It was also demonstrated that young people with weak motivation for health-oriented physical activity encountered a three times greater risk (OR=2.96, 95% CI 2.36-3.72, p=0.000) of perceiving accumulated barriers in comparison to adolescents with strong motivation.

In the analyses conducted separately in groups of young people with different levels of motivation for undertaking health-oriented physical activity, it was demonstrated that the excess of body weight turn out to be an important predictor of the perception of a multitude of barriers to physical activity only in adolescents with weak motivation. In case of young people with strong and average health-oriented motivation, the excess of body weight ceased to be significant for the perception of accumulated barriers to health-oriented physical activity (tab. VI).

## Discussion

Numerous psychological factors which can be potential predictors of insufficient physical activity in adolescents were examined in the last two decades on the basis of the social learning theory [17, 22]. These also include the perceived facilities and barriers to undertaking physical activity. Moreover, it is an important direction in the research on adolescents with overweight and obesity.

The first objective of this paper was to identify barriers to physical activity in adolescents aged 13-16 with different BMI status. It was found that three out of five of the investigated barriers were indeed more often listed by overweight and obese adolescents than by their peers with normal weight. These barriers were among the so-called internal barriers. They resulted from the self-limitations of adolescents, relating primarily to lack of skills, energy and willpower. This is important information, because such barriers are the proof of low internal motivation for engaging in physical activity among young people with overweight and obesity [23]. Body weight was a more important factor differentiating the perception of the barriers in boys than in girls. This was probably caused by a greater percentage of girls perceiving barriers to physical activity among the study participants. In another study relating to this group of young people that did not take into account body weight, it was concluded that girls were twice as likely to perceive the barriers to physical activity in comparison with boys [24].

Another objective of the study was to investigate the extent to which the perception of the barriers to physical activity changes in a group of adolescents depending on body weight and differences in the level of health-oriented motivation to engage in physical activity.

In comparison to adolescents with a healthy body weight, young people with overweight and obesity were not motivated to physical activity by the need to get in good shape and to be good at sport; stronger motivation was the need to control weight, though a statistically significant correlation occurred only in boys.

It can be presumed that among girls dietary efforts play a greater role in controlling body weight than physical activity [25]. Polish research on eating behaviours and body weight control methods in older adolescents (aged 16-18) has found that girls follow diets three times more often than boys (or engage in other behaviour aimed at weight loss). An exception is the intensification of physical exercise noted in a greater number of boys. A proper diet (including expert-supervised diets) is followed by 60% of boys and even 90% of girls [26].

The results of the multilevel logistic regression model applied in our study showed a significant influence of health-oriented motivation on the perception of barriers to physical activity by overweight and obese adolescents. In the model oriented on different levels of motivation for physical activity, it was concluded that the excess body weight as a predictor of the perception of accumulated barriers to physical activity remained in the model only in cases of weak health-oriented motivation. In adolescents with strong and average motivation for physical activity, the excess body mass ceased to be significant for the perception of accumulated barriers to physical activity. According to Korean authors, the BMI alone explained only 17% of the variability of physical activity in adolescents with overweight and obesity, however, after adding a component compliant with the self-determination theory motivation for physical activity, the percentage rose to 49% [27].

It can be concluded that young people with excessive body weight and strong health-oriented motivation perceived barriers to physical activity similarly to their peers with normal weight. Thus, motivation was a key element, shaping overweight and obese young people’s physical activity by changing their perception of the barriers to physical activity.

Both in comprehensive preventive measures and in the treatment of obesity in adolescents, physical education (PE) teachers have a special role to play alongside parents. We might still be far away from a situation where students have an opportunity to participate in a mandatory PE lesson tailored to their individual needs, abilities and expectations. However, teachers’ help might prove to be important as a breakthrough in increasing their motivation, especially if it is health-oriented, to undertake and maintain an appropriate level of physical activity [28, 29].

## Conclusions

Recognising the barriers to physical activity in overweight and obese adolescents might facilitate adopting an individual approach in PE classes, as well as devising effective intervention programmes aimed at ones with excessive body weight.

In overcoming the barriers to physical activity in overweight and obese adolescents, one should aim to comprehensively reduce body weight and support health-oriented motivation for physical activity.

The greatest chances of success are envisaged for the forms of assistance which include working with overweight young people and are oriented both towards the individual and the environment, supporting health-oriented motivation for physical activity.
